# Application of non-invasive preimplantation genetic screening for aneuploidy through spent embryo culture media analysis at 48 and 54 hours after embryo cleavage

**DOI:** 10.3389/bjbs.2026.15905

**Published:** 2026-06-09

**Authors:** Chonthicha Satirapod, Pornsri Niransuk, Siriluk Tantanavipas, Insee Sensorn, Wasun Chantratita, Objoon Trachoo

**Affiliations:** 1 Department of Obstetrics and Gynecology, Faculty of Medicine Ramathibodi Hospital, Mahidol University, Bangkok, Thailand; 2 Center of Medical Genomics, Faculty of Medicine Ramathibodi Hospital, Mahidol University, Bangkok, Thailand; 3 Department of Medicine, Faculty of Medicine Ramathibodi Hospital, Mahidol University, Bangkok, Thailand; 4 Department of Medical Genetics, College of Health Sciences, VinUniversity, Hanoi, Vietnam; 5 Center for Global Health, Perelman School of Medicine, University of Pennsylvania, Philadelphia, PA, United States

**Keywords:** aneuploidy, non-invasive, preimplantation genetic testing for aneuploidy (PGT-A), spent culture media, trophectoderm biopsy

## Abstract

**Background:**

This study aims to assess the effectiveness of non-invasive preimplantation genetic testing (niPGT) for detecting aneuploidy at two different time points, 48- and 54-hour following the cleavage stage of embryo development.

**Methods:**

A cohort of 15 infertile women was enrolled, involving a total of 58 embryos. All embryos underwent sequential culture media procedures and received assisted hatching during the cleavage stage on Day 3. After biopsy examination, conducted either 48 or 54 hours post cleavage, spent culture media (SCM) were gathered and processed for the amplification and quantification of cell-free DNA. This was followed by low-pass whole genome sequencing.

**Results:**

The cell-free DNA content within SCM remained consistent across both time points post cleavage. The accuracy of niPGT in ploidy detection, in comparison to trophectoderm biopsy, was 53.71%. No significant distinction in ploidy detection accuracy was observed between SCM collected from embryos at 48 hours versus those at 54 hours post cleavage. The overall accuracy for sex determination reached 79.63%.

**Conclusions:**

The concentration of cell-free DNA within SCM was found to be consistent at both 48- and 54-hours after embryo cleavage. However, the accuracy of ploidy determination, when contrasted with the conventional trophectoderm biopsy, did not yield satisfactory outcomes.

## Introduction

Aneuploidy is a concern that can significantly impact the success of *in-vitro* fertilization (IVF), particularly for women of advanced age who face an elevated risk of miscarriage or aberrant pregnancy occurrences attributed to chromosomal irregularities. Previous research has revealed that fetal tissue sampled from first-trimester miscarriages, encompassing both natural pregnancies and those conceived by assisted reproductive technologies, exhibited an overall rate of abnormal fetal karyotypes ranging from 50% to 67.8% [1, 2]. Preimplantation genetic testing for aneuploidy (PGT-A) represents a technique employed to promptly identify chromosomal irregularities in embryos and to opt for the transfer of individual euploid embryos, consequently leading to elevated success rates and a decreased timeframe for achieving pregnancy. This approach also contributes to diminishing the probabilities of chromosomal disorders, multiple pregnancies, and maternal complexities. PGT-A is recommended for patients encountering advanced maternal age, recurrent implantation failure, repeated miscarriages, and substantial male factor infertility [3]. The sources of the samples gathered for DNA analysis encompass embryos at either the cleavage or blastocyst stage. The PGT-A process permits an overarching error rate ranging from 1% to 3% and necessitates a comprehensive dialogue with the couple regarding the potential for misdiagnosis [3]. In the context of contemporary IVF, the paradigm of PGT has transitioned towards blastocyst biopsy. This methodology involves the biopsy of the trophectoderm (TE) on days 5–6, mainly due to its minimal influence on embryo implantation potential and viability [4]. This stands in contrast to the diminished rates of implantation associated with biopsies conducted at the cleavage stage [5].

Although PGT-A stands as a customary technique for embryo diagnosis, it remains an invasive procedure necessitating skilled embryologists and robust technical expertise. The long-term consequences of embryonic biopsy are still unclear [6]. Maternal and neonatal outcomes can be influenced by this procedure, including an elevated risk of pre-eclampsia and fetal growth restriction [7, 8]. In tandem with the advancement of PGT-A, methodologies were developed for generating substantial DNA quantities through whole genome amplification (WGA) and subsequent next-generation sequencing ^2^, offering a cost-effective and swift outcome [9, 10]. Non-invasive preimplantation genetic testing (niPGT) for aneuploidy emerged employing WGA to enable consistent DNA analysis within embryo culture media. Spent culture media (SCM) derived from developing human embryos can be conveniently collected and is less intricate than TE biopsy. A prior investigation demonstrated the feasibility of using cell-free DNA (cfDNA) within the culture medium to identify potential α-thalassemia and sex chromosome abnormalities in embryos [11]. Experimental data indicated comparable chromosomal outcomes between niPGT-A and TE biopsy analyses [6]. Following initial proof-of-concept studies [12], the use of cfDNA in SCM as a non-invasive alternative for PGT-A has been studied and extensively published. Previous studies report high sensitivity for the detection of chromosomal abnormalities, with concordance rates between SCM alone and TE biopsies, ranging from 62.1% to 88.9% [13–15]. Alternatively, minimally invasive approaches, combining SCM with blastocoel fluid, have also been utilized to assess concordance [16].

Furthermore, studies comparing SCM directly to the inner cell mass (ICM) [17, 18] or the whole blastocyst [19, 20] have demonstrated concordance rates ranging from 32% to 86%, suggesting that SCM may better represent the genetic status of the entire embryo rather than just the TE.

Despite these promising findings and recent systematic reviews showing improved overall concordance, specificity remains variable, and many studies are limited by small sample sizes [21, 22]. A significant knowledge gap persists due to inconsistent outcomes, technical heterogeneity, and variation in sequencing platforms. Most importantly, there is still no definitive consensus on the optimal time point for SCM collection, which remains a critical variable affecting cfDNA yield and diagnostic reliability.

The duration of embryo exposure to culture media correlates with the quantity of DNA present and the likelihood of successful DNA amplification from the media. Prior studies largely omitted a precise timing for SCM collection. Nevertheless, recent research indicated a positive association between increased DNA content in SCM and prolonged embryo exposure duration [15, 23]. Furthermore, extended periods of culture media exposure to embryos, extending up to day 7, may exhibit a correlation with DNA quantity and chromosome analysis outcomes [13, 24]. Within our ART unit, TE biopsy is routinely conducted on day 5, lacking a protocol for non-invasive techniques and the optimal timing of sample collection to ensure optimal DNA quantity and analytical accuracy. This study aims to assess the effectiveness of niPGT in comparison to TE biopsy using SCM obtained from varying time points during embryo culture.

## Materials and methods

### Ethics approval

Approval for this study was obtained from the Ethics Clearance Committee on Human Rights Related to Research Involving Human Subjects, Faculty of Medicine, Ramathibodi Hospital, Mahidol University, ID MURA2020/698. All patients enrolled in the study provided informed consent during the period from August 2020 to February 2021. Recruitment of participants was facilitated based on recommendations from nurses and physicians, resulting in the inclusion of 15 patients.

### Population

The study targeted women aged between 30- and 45-year experiencing infertility and undergoing IVF. Informed consent was secured for the utilization of residual culture medium. Embryos cultured for less than 5 days and those graded below the early blastocyst stage were excluded from the study.

### Study protocol

#### Intracytoplasmic sperm injection and embryo culture

**FIGURE 1:**
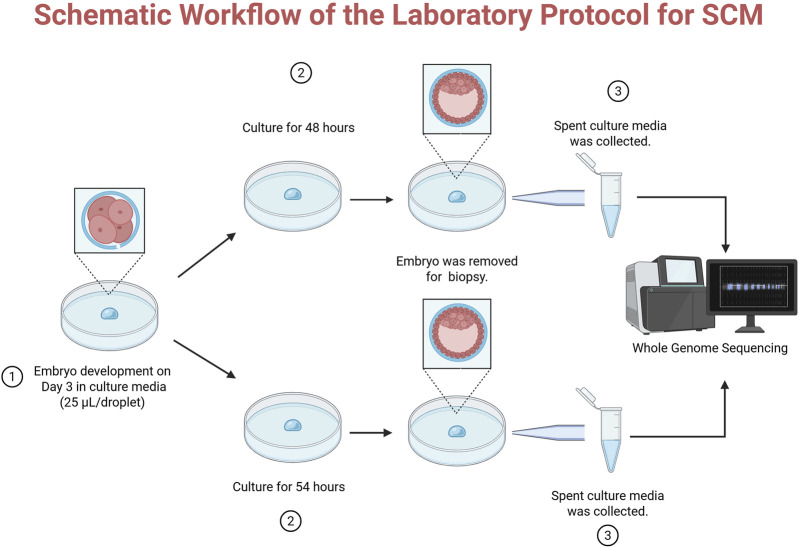
Schematic workflow of the non-Invasive preimplantation genetic testing laboratory protocol. Created in BioRender. Niransuk, P. (2026) https://BioRender.com/qkwbbsg.

The intracytoplasmic sperm injection (ICSI) procedure adhered to a standard protocol. Following oocyte retrieval, the oocyte was placed within a Petri dish in an environment of 5% CO2 at 37 °C for a period of 2–4 h. Subsequently, the oocyte was disengaged from the cumulus-oophorus complex employing hyaluronidase (FertiPro, Beernem, Belgium). ICSI was carried out on metaphase II oocytes, positioned in 25–30 µL droplets of fertilization medium (Cook Medical, Bloomington, IN). Fertilization status was assessed 16–18 h post ICSI. Embryos exhibiting two pronuclei were transferred to 25–30 µL droplets of cleavage culture medium (Cook Medical), subsequently covered with paraffin oil (LifeGlobal, Guilford, CT). On the third day post fertilization, embryo development stage was evaluated in accordance with the Gardner and Schoolcraft morphology classification. Embryos at day 3 post-fertilization were allocated to either the 48-h or 54-h culture group. All embryos from a given patient were biopsied at the same assigned time point. Each patient contributed embryos to only one group, with no overlap between the 48-h and 54-h groups. Employing a laser technique beneath an inverted microscope (Nikon Eclipse Ti, Nikon, Tokyo, Japan), assisted hatching was performed, followed by individual transfer of embryos to 25 µL droplets of blastocyst medium (Cook Medical). Each culture incorporated negative control culture medium to ensure quality control (Figure 1).

#### Washing medium preparation

Phosphate buffered saline (PBS, pH 7.4, Kitazato, Shizuoka, Japan) was freshly prepared on the same day as the SCM collection. Sterile Petri dishes (polystyrene, 50 × 9 mm, Falcon, Thermo Fisher Scientific, Waltham, MA) were allocated twelve to fifteen drops of PBS, which served as the washing medium for pipette tips.

### Spent culture medium collection

On day 5, 48 h following the cleavage stage, embryos were subjected to re-evaluation regarding their developmental stage. Only embryos at the early blastocyst stage or beyond were included in the study. The early blastocyst stage was defined according to the Gardner and Schoolcraft blastocyst grading system, as expansion stage 2, characterized by the presence of a visible blastocoel cavity occupying less than 50% of the embryo volume. For TE biopsy, embryos were first transferred from the original culture drop to a fresh biopsy drop using an embryo handling pipette to minimize potential contamination. TE biopsy was then performed in the biopsy drop under standard micromanipulation conditions. Following embryo removal, SCM was collected from the original culture drop. To collect each SCM sample, a fresh and sterile pipette tip was used, which had been rinsed with PBS to reduce contamination. Approximately 25 µL of SCM was transferred into a sterile PCR tube. As a control, SCM samples were also collected from culture drops without embryos. All procedures were performed promptly to preserve DNA integrity. Subsequent to collection, all samples were promptly stored at −20 °C. The specimens collected on day 5 and 54 h post cleavage stage underwent the same process as described above. For shipment to the genomic laboratory, the samples were dispatched with dry ice.

### Whole-genome DNA amplification of spent culture medium

The procedure adhered to the Non-Invasive PGT kit protocol (PG-Seq, PerkinElmer, Waltham, MA). In the first round, 6 µL of each SCM sample was introduced into a sterile PCR tube, followed by the addition of 21 µL of PCR master mix to each sample. The same process was undertaken for the culture medium droplets without embryos on the same plate, serving as a negative control to monitor contamination. Following thorough mixing and a brief centrifugation step, the samples were incubated in a thermal cycler programmed according to established standards (PerkinElmer). The total cycling duration was approximately 1.5 h. Subsequently, the PCR tubes containing round one samples were placed in a cold block to facilitate round two PCR. This stage involved the addition of 46 µL of PCR master mix per sample, and after a brief centrifugation, the final mixtures underwent amplification in a thermocycler programmed for 30 min. The resulting WGA products were subsequently purified and stored at −20 °C.

### Quantification of whole genome amplification

Quantification of whole genome amplification (WGA) products was performed using the Qubit fluorometric system (Thermo Fisher Scientific) in accordance with the manufacturer’s protocol.

### Ultra-low-pass whole-genome sequencing

**FIGURE 2:**
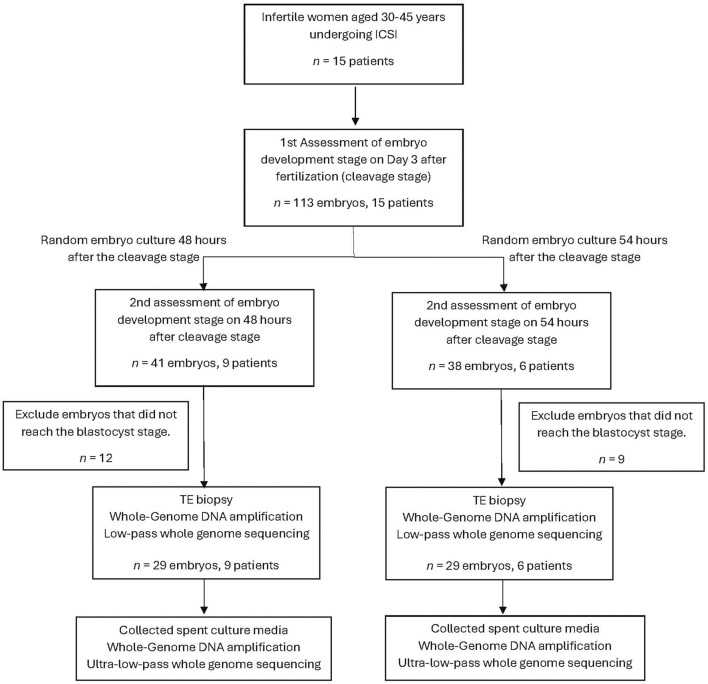
Study design and workflow for embryo culture, assessment, and genetic analysis.

Following confirmation of adequate DNA yield, ultra–low-pass whole-genome sequencing was performed using the Ion OneTouch System, in accordance with the manufacturer’s instructions. The estimated sequencing depth of this platform was approximately 0.01× (400,000–500,000 reads/sample). Sequencing generated FASTQ files, which were subsequently aligned to produce BAM files for downstream analysis. The results were independently interpreted by two embryologists (Figure 2).

### Pre-implantation genetic screening for aneuploidy in trophectoderm-biopsied samples

PGT-A of trophectoderm (TE) biopsy samples was performed at the Reproductive Genomic Service Laboratory, Ramathibodi Hospital, in accordance with the manufacturer’s protocol for the Ion ReproSeq PGS Kit (Thermo Fisher Scientific, Waltham, MA), in combination with a low-pass whole-genome sequencing workflow (Figure 2).

### Statistical analysis

The statistical analysis was conducted using Stata, Version 14.0 (StataCorp, College Station, TX). Continuous variables were presented as mean with standard deviation (SD) or median with interquartile range (IQR) based on the distribution characteristics. Unpaired t-tests or Mann–Whitney-U tests were employed to compare continuous variables between different groups. Fisher’s exact tests were utilized for qualitative comparison of independent data between two groups. A significance level of *p* ≤ 0.05 was adopted to denote statistical significance.

## Results

### Demographic data

A total of 58 embryos were procured from 15 patients at the Reproductive Endocrinology and Infertility Unit, Faculty of Medicine, Ramathibodi Hospital, spanning the period from August 2020 to February 2021. The mean age of patients who contributed embryos for analysis at both 48 and 54 h post cleavage-stage was 36.4 ± 3.8 and 36.2 ± 2.5 years, respectively (*p* = 0.72). Most indications for PGT-A were related to advanced maternal age and a history of recurrent miscarriages. Embryos underwent TE biopsy either 48 or 54 h after the cleavage stage. Embryos underwent TE biopsy either 48 or 54 h after the cleavage stage. The outcomes demonstrated a detection of 37.9% and 31% euploid embryos in samples obtained at 48 and 54 h post cleavage-stage, respectively (Table 1).

**TABLE 1:** Baseline demographic characteristics.

Variables	48h after cleavage stage	54h After cleavage stage	*p* value
Number (total)	29	29	NA
Number of patients	9	6	0.69
Age (years old) (mean ± SD)	36.4 ± 3.8	36.2 ± 2.5	0.72
Type of infertility (n, %) Primary Secondary	6 (66.7%) 3 (33.3%)	4 (66.7%) 2 (33.3%)	1.00
Cause of infertility (n, %) Female Male Combined	6 (66.7%) 2 (22.2%) 1 (11.1%)	5 (83.3%) 1 (16.7%) 0 (0.0%)	0.78
History of miscarriage (n, %) No Yes ( ≥ 1 times)	7 (77.8%) 2 (22.2%)	4 (66.7%) 2 (33.3%)	0.61
Duration of infertility (years) (median, min-max)	2.0 (1–3)	3.0 (1–4)	0.39
Indication for PGT-A	Advanced age Recurrent pregnancy loss	Advanced age Recurrent pregnancy loss Previous DFIU	NA
Protocol for ovarian stimulation	Antagonist protocol	Antagonist protocol	NA
TE biopsy outcome - Euploid - Aneuploid - Mosaicism	(29) 11 (37.9%) 16 (55.2%) 2 (6.9%)	(29) 9 (31.0%) 15 (51.7%) 5 (17.3%)	0.58
TE biopsy Undetermined outcome	No	No	NA

*p*-value was calculated using Fisher’s exact test for categorical variables. For continuous variable, independent t-test was used for data presented as mean ± SD, and the Mann-Whitney U test was used for data presented as median (min-max). *p*-value < 0.05 was considered statistically significant.

PGT-A, preimplantation genetic testing for aneuploidy; TE, trophectoderm; DFIU, dead fetus *in utero*; NA, not applicable.

### DNA quantification between 48- and 54-hours

All SCM samples were subjected to DNA amplification and sequencing. The success rate of PCR amplification at both time points stood at 93.1%. The median DNA quantities were 27.8 ng/mL (IQR 18.7–35.6 ng/mL) and 25.8 ng/mL (IQR 12.6–35.2 ng/mL) at 48 and 54 h, respectively (Table 2). The quantities of cfDNA exhibited similarity between the SCM samples from both groups (*p* = 0.7). Amplification of negative control samples (media drops lacking embryos, cultured under equivalent conditions) for the 48 and 54-h groups resulted in DNA quantities of 0.92 ng/mL and 0.82 ng/mL, respectively (*p* = 0.89). The amplification failure rate for both groups was comparable (6.9%) and demonstrated no correlation with embryo grading or duration of embryonic exposure.

**TABLE 2:** Comparison of DNA quantity.

DNA measurement	48 h after cleavage stage	54 h after cleavage stage	*p*-value
DNA quantity [ng/mL, median (IQR)]	27.8 (18.7–35.6)	25.8 (12.6–35.2)	0.70
Failed amplification	2 (6.9%)	2 (6.9%)	1.00
Negative control [ng/mL DNA, median (IQR)]	0.92 (0.77–1.15)	0.82 (0.81–1.25)	0.89

IQR, interquartile range.

### Evaluation of niPGT efficacy

SCM results were categorized based on ploidy (euploid and non-euploid) and sex chromosome status (male or female), subsequently juxtaposed with molecular findings acquired from TE biopsy. Terminology of ploidy concordance between SCM and TE biopsy results was defined ad and agreement in the overall ploidy diagnosis (i.e., euploid-euploid or aneuploid-aneuploid), regardless of whether the specific affected chromosomes in the aneuploid samples perfectly matched. Similarly, sex concordance was defined as an agreement in the predicted sex chromosomes between the two methods. Our results showed that the overall ploidy concordance rate between niPGT and TE biopsy was 53.7% (29/54). The ploidy concordance rate was comparable between the 48-h and 54-h groups (48.2% vs. 59.3%, respectively; *p* = 0.41). Similarly, the overall sex concordance rate was 79.6% (43/54), with no significant difference between the 48-h and 54-h groups (74.1% vs. 85.1%, respectively; *p* = 0.31) (Table 3).

**TABLE 3:** Ploidy and sex determination accuracy of non-invasive preimplantation genetic testing *versus* trophectoderm biopsy.

Detection accuracy	48 h after cleavage	54 h after cleavage	Overall	*p*-value (48 h vs. 54 h)
Ploidy detection accuracy - Euploid - Non-euploid	48.15% 25.93% 22.22%	59.25% 11.10% 48.15%	53.71% 18.52% 35.19%	0.41
Sex detection accuracy	74.07%	85.1%	79.63%	0.31

The efficacy of niPGT in detecting ploidy status, compared with TE biopsy, was assessed. Sensitivity and specificity of niPGT in identifying euploid embryos were found to be 55.6% and 52.8%, respectively. The positive predictive value and negative predictive value of niPGT for discerning euploid embryos were 37% and 70.4%, respectively. Comparison between the 48-h and 54-h group showed no significant differences in these diagnostic metrics (Table 4). Nevertheless, niPGT using SCM collected at 54-h demonstrated a trend toward a higher NPV for correctly excluding aneuploid embryos.

**TABLE 4:** Performance comparison of non-invasive preimplantation genetic testing *versus* trophectoderm biopsy.

Parameter	48 h after cleavage	54 h after cleavage	niPGT (overall)	p-value (48 h vs. 54 h)
Sensitivity	63.6% (30.8–89.1)	42.9% (9.9–81.6)	55.6% (30.8–78.5)	0.39
Specificity	37.5% (15.2–64.6)	65% (40.8–84.6)	52.8% (35.5–69.6)	0.10
PPV	41.2% (18.4–67.1)	30% (6.7–65.2)	37% (19.4–57.6)	0.56
NPV	60% (26.2–87.8)	76.5% (50.1–93.2)	70.4% (49.8–86.2)	0.37

PPV, positive predictive value; NPV, negative predictive value.

## Discussion

The present study aimed to evaluate the optimal timing for SCM collection in the context of niPGT. In our institute, embryos undergo laser-assisted hatching on day 3, followed by sequential culture and TE biopsy on day 5. We therefore compared two SCM collection time points: 48 and 54 h post–cleavage stage.

Our hypothesis was that extending the culture duration by an additional 6 h, from 48 to 54 h, could enhance cfDNA yield by allowing increased cellular turnover and DNA release into the culture medium, thereby reducing amplification failure. This rationale is supported by morphokinetic studies demonstrating that aneuploid embryos exhibit delayed developmental milestones compared to euploid embryos, including initiation of compaction, onset of blastulation, and time to full blastocyst formation, with delays often exceeding 6 h [25]. Such temporal asynchrony in embryonic development suggests that biological processes associated with cell turnover and cfDNA release may also vary over short time intervals [25].

Based on this evidence, we hypothesized that a modest extension of the culture period would increase the likelihood of capturing sufficient embryonic cfDNA in SCM, particularly in embryos with delayed developmental kinetics, thereby improving downstream diagnostic performance.

However, our study showed the quantity of cfDNA was not significantly different between the 48-h and 54-h culture groups. While the concordance of ploidy detection trended higher in the 54-h group, the overall accuracy remained limited at 53.7%. Furthermore, the diagnostic performance (sensitivity, specificity, PPV, and NPV) showed no significant differences between the two time points and the wide confidence intervals observed in our data indicate that our subgroup analysis was statistically underpowered. These findings may suggest that collecting SCM at a later developmental stage may be necessary before niPGT can be considered for clinical application. While the absolute optimal timing for SCM collection remains a subject of ongoing investigation, we acknowledge that Day-6 SCM sampling has currently demonstrated higher cfDNA yields and superior concordance rate [26, 27]. Ultimately, our present finding largely confirms existing knowledge regarding the limitation of early SCM sampling.

This study systematically examined potential disparities in culture duration for sample collection, subsequent DNA quantities, and PGT-A outcomes. Rigorous protocols were enacted to prevent contamination, an assertion supported by the results from negative control medium (<1.5 ng/mL DNA). The approach adopted here yielded a commendable amplification success rate of 93.1%, a figure notably higher compared to prior investigations [28, 29]. However, it is important to note that the amplification success rate from SCM can vary significantly across different laboratories and protocols. While some studies reported low rates [27, 30], other groups have successfully published much higher amplification rates, reaching up to 100% [31, 32].

Notably, however, there was no observable divergence in the quantity of cell-free DNA within SCM samples obtained from the 48 and 54-h groups. This outcome might be attributed to the limited sample size or the relatively brief interval between the varied culture times studied. The niPGT results in our study showed overall ploidy concordance rate was limited at 53.7%. However, the precision for fetal sex prediction was relatively higher compared to the overall aneuploidy concordance rate, achieving 79.6%. Interestingly, these ploidy results stand in contrast with prior studies that suggested a superior performance of niPGT in identifying embryo aneuploidy [33].

Within the current study, the accuracy of sex determination exhibited a discernible positive trend as the incubation period was extended. In select scenarios, such as couples at risk of X-linked disorders, niPGT for sex determination could potentially hold value for informed reproductive planning. Certain IVF clinics have already adopted niPGT employing techniques like multiple annealing and looping-based amplification cycles, subsequently complemented by next-generation sequencing for clinical utilization, resulting in successful pregnancies [6]. However, it is our view that this alternative approach necessitates further refinement and validation prior to its integration into routine clinical practice.

Moreover, niPGT holds promise in optimizing the outcomes of preimplantation genetic testing for monogenic disorders (PGT-M). Our institution has garnered a decade of experience in conducting PGT-M, specifically for hemoglobinopathy and various rare genetic disorders [34, 35]. Our established protocol involves the use of double TE biopsy techniques, given the distinct WGA templates essential for PGT-M and PGT-A, necessitating two sample pools for concurrent WGA preparation. Employing these double techniques resulted in an overall successful live birth rate of 53.33% for couples at risk for beta thalassemia/Hb E disease [35]. However, it is essential to acknowledge that this protocol may contribute to potential embryo damage and influence implantation rates [4, 5]. Consequently, adopting niPGT offers a clear advantage by eliminating the need for an additional embryo biopsy for PGT-A. In cases requiring PGT-M, it reduces embryo manipulation to a single biopsy, while still enabling concurrent aneuploidy screening through niPGT.

As discussed, niPGT bears potential advantages for clinical applications. This non-invasive avenue, entailing SCM sample collection, demands less technical expertise than traditional TE biopsy [36]. Our assertion is that this alternative methodology provides a viable option for IVF laboratories where a skilled embryologist for biopsy procedures is lacking, and furthermore, it might circumvent potential religious and legal dilemmas tied to embryo manipulation.

At present, the question remains whether the DNA found within culture media truly represents the authentic karyotype of the embryo. Theoretical speculation suggests that SCM contains apoptotic cells from both the inner cell mass and TE, a phenomenon that could arise from either abnormal cells or the inherent embryonic cellular clearance process. Within this study, a considerable proportion of embryos failing to yield amplified DNA from SCM exhibited a normal karyotype. This phenomenon could be attributed to technical errors or the possibility that euploid embryos release lesser cell-free DNA into culture media. Moreover, SCM might also harbor cumulus cells, previously identified as a significant contributor to maternal DNA contamination [37].

Furthermore, large multicenter and prospective studies have demonstrated that cfDNA represents both ICM and TE, showing high ploidy concordance of cfDNA with ICM and with TE, 78%–90% [17, 38]. These high concordance rates are typically achieved when maternal contamination, such as from cumulus cells, is tightly controlled and proper protocol are strictly followed. In contrast, some studied have reported the lower concordance rate (20%–60%) and poor DNA quality, which are often attributed to maternal contamination or variations in laboratory protocols [19, 37].

Our study has several methodological strengths that enhance the reliability of the baseline data. These include strict inclusion criteria, wherein only embryos reaching the early blastocyst stage or higher were analyzed, as well as the use of highly consistent ICSI and embryo culture protocols. Furthermore, the rigorous use of negative medium controls allowed for accurate assessment and exclusion of environmental background contamination.

However, this study has several limitations. First, it was conducted in a single center with a small sample size, which may limit the generalizability of the findings. Second, our study lacks randomization or strict matching regarding the exact embryo development stages and morphological grading. Variation in embryo development kinetics could influence the quantity and quality of cfDNA released into spent culture medium. Another important limitation is that maternal DNA contamination in the SCM was not quantitatively assessed (e.g., via short-tandem repeat analysis or methylation profiling), which could be a primary cause of the discordant results between niPGT and TE biopsies. Future studies should incorporate embryo grading matching and robust maternal DNA quantification techniques to ensure the accuracy and reliability of niPGT results.

## Conclusion

The quantity of cell-free DNA present in SCM derived from embryo cultures demonstrated equivalence at both 48 and 54 h following the embryo cleavage stage. Although the accuracy of ploidy status determination through niPGT was found to be suboptimal, with only approximately half of the tested samples yielding accurate outcomes when compared with PGT-A results from TE biopsy, there was a notable potential for enhanced sex chromosome identification using niPGT. This innovative non-invasive approach is currently in the developmental phase, offering the advantage of reduced technical demands on embryologists and lowered costs. Particularly in regions where ethical and legal constraints prohibit embryo biopsy, this technique presents a promising solution. To summarize, a deeper exploration of niPGT is imperative to pave the way for its potential future clinical implementation.

## Summary table

### What is known about this subject

- PGT-A is widely used but invasive, requiring technical expertise and potentially associated with adverse maternal and neonatal outcomes.
- niPGT using spent culture media (SCM) has emerged as a non-invasive alternative, enabled by WGA and NGS technologies.
- Reported concordance between SCM and TE biopsy is variable (62%–89%), with no consensus on the optimal timing of SCM collection.

### What this paper adds

- Demonstrates modest concordance between niPGT and TE biopsy for ploidy (53.7%) and higher concordance for sex determination (79.6%).
- Shows no significant differences in diagnostic performance between SCM collected at 48 vs. 54 h post-cleavage.
- Identifies a trend toward higher negative predictive value at 54 h, suggesting improved potential for excluding aneuploid embryos.

## Concluding statement

This work represents an advance in biomedical science because it refines the optimal timing of SCM collection and highlights the potential of niPGT, particularly for non-invasive exclusion of aneuploid embryos.

## Data Availability

Publicly available datasets were analyzed in this study. This data can be found here: Genome assembly GRCh37, National Center for Biotechnology Information. https://api.ncbi.nlm.nih.gov/datasets/v2/genome/accession/GCF_000001405.13/download?include_annotation_type=GENOME_FASTA&include_annotation_type=GENOME_GFF&include_annotation_type=RNA_FASTA&include_annotation_type=CDS_FASTA&include_annotation_type=PROT_FASTA&include_annotation_type=SEQUENCE_REPORT&hydrated=FULLY_HYDRATED.
